# Effect of Exercise on Fatty Acid Metabolism and Adipokine Secretion in Adipose Tissue

**DOI:** 10.3389/fphys.2019.00026

**Published:** 2019-01-28

**Authors:** Adriana Mika, Filippo Macaluso, Rosario Barone, Valentina Di Felice, Tomasz Sledzinski

**Affiliations:** ^1^Department of Pharmaceutical Biochemistry, Faculty of Pharmacy, Medical University of Gdańsk, Gdańsk, Poland; ^2^Department of Environmental Analysis, Faculty of Chemistry, University of Gdańsk, Gdańsk, Poland; ^3^Department of Biomedicine, Neurosciences, and Advanced Diagnostic (Bi.N.D.), University of Palermo, Palermo, Italy; ^4^Euro-Mediterranean Institute of Science and Technology, Palermo, Italy; ^5^SMART Engineering Solutions & Technologies (SMARTEST) Research Center, eCampus University, Palermo, Italy

**Keywords:** exercise, adipose tissue, fatty acid, adipokine, myokine, adipose tissue beiging

## Abstract

Increased physical activity is an optimal way to maintain a good health. During exercise, triacylglycerols, an energy reservoir in adipose tissue, are hydrolyzed to free fatty acids (FAs) which are then released to the circulation, providing a fuel for working muscles. Thus, regular physical activity leads to a reduction of adipose tissue mass and improves metabolism. However, the reduction of lipid reservoir is also associated with many other interesting changes in adipose tissue FA metabolism. For example, a prolonged exercise contributes to a decrease in lipoprotein lipase activity and resultant reduction of FA uptake. This results in the improvement of mitochondrial function and upregulation of enzymes involved in the metabolism of polyunsaturated fatty acids. The exercise-induced changes in adipocyte metabolism are associated with modifications of FA composition. The modifications are adipose tissue depot-specific and follow different patterns in visceral and subcutaneous adipose tissue. Moreover, exercise affects adipokine release from adipose tissue, and thus, may mitigate inflammation and improve insulin sensitivity. Another consequence of exercise is the recently described phenomenon of adipose tissue “beiging,” i.e., a switch from energy-storing white adipocyte phenotype to thermogenic FA oxidizing beige adipocytes. This process is regulated by myokines released during the exercise. In this review, we summarize published evidence for the exercise-related changes in FA metabolism and adipokine release in adipose tissue, and their potential contribution to beneficial cardiovascular and metabolic effects of physical activity.

## Introduction

**Figure 1:**
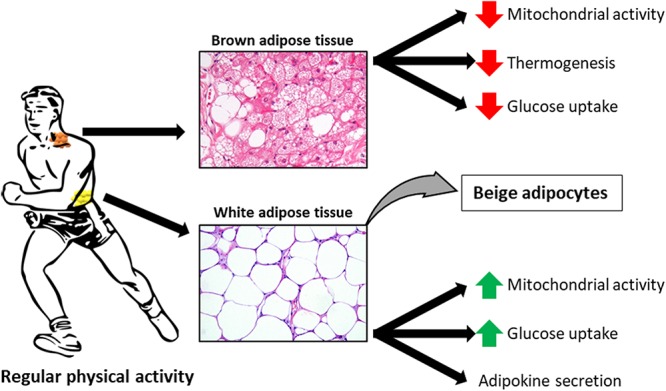
Exercise-induced adaptations to white adipose tissue (WAT), brown adipose tissue (BAT) and beige adipocytes. Histological sections of WAT and BAT are stained with haematoxylin and eosin.

In the 21st century, when obesity is recognized as a civilization-related, economic and social burden and the numbers of obese and overweight individuals still increase, we need new strategies to prevent and treat those conditions. Since excess body weight results from an imbalance between energy intake and energy expenditure (Jakicic and Otto, 2005), one way to maintain a correct body weight is to stimulate lipid catabolism through increased physical activity. Appropriately designed training simulates lipolysis, i.e., the hydrolysis of triacylglycerols stored in adipose tissue (AT), which results in the release of free fatty acids (FFAs) to circulation and their oxidation in muscles and other tissues. Elevated blood concentration of FFAs, observed in obesity and metabolic syndrome, is an adverse condition that may lead to lipotoxicity and ectopic deposition of lipids in other tissues (Mika and Sledzinski, 2017). Thus, efficient uptake and oxidation of FFAs in working muscles are critical for maintaining their normal blood levels. Moreover, exercise contributes to an increase in the number of mitochondria in white AT (WAT) and stimulates the expression of brown adipocyte-specific genes, which leads to “beiging” of WAT and amelioration of glucose intolerance induced by a high-fat diet (Sutherland et al., 2009; Xu et al., 2011; Roberts et al., 2014) (Figure 1). These effects of exercise on AT are associated with significant changes in metabolism and composition of fatty acids (FAs), the main components of adipocytes. Aside from the storage of triacylglycerols, AT acts also as an endocrine organ, releasing many biologically active substances referred to as adipokines. Exercise may also modulate the endocrine function of AT. There are three types of AT: WAT, located subcutaneously and viscerally, brown adipose tissue (BAT), and beige AT formed as a consequence of white adipocyte “beiging,” i.e., their phenotypic and metabolic transition to cells similar to brown adipocytes. WAT, abundant in both humans and rodents, is primarily responsible for triacylglycerols storage and release of various adipokines into the blood. While in rodents BAT forms a large interscapular depot, as well as smaller depots in other locations, its existence in humans, around the neck, spine and major blood vessels, has been demonstrated quite recently. BAT, rich in mitochondria, is primarily responsible for thermogenesis (Lehnig and Stanford, 2018). Recent studies in humans and in rodents have identified controversial results in BAT activity in response to regular physical exercise. In trained humans, Vosselman et al. (2015) and Motiani et al. (2017) have observed a decrease in BAT activity (mitochondrial activity, glucose uptake, and thermogenesis, Figure 1). In this review, we discuss the exercise-induced changes in the composition and metabolism of FAs in AT, with particular emphasis on AT depot-specific differences.

## Effect of Exercise on FA Composition in AT

The release of FAs from adipocytes to deliver them to working muscles contributes to changes in the amount and composition of AT lipids. However, these effects were shown to depend on the exercise intensity (Nikolaidis and Mougios, 2004). Some studies demonstrated that low-intensity endurance training leads to maximal lipid oxidation, but available evidence in this matter is inconclusive (Romain et al., 2012). Triacylglycerols are the major class of lipids, representing up to 90–99% of all AT lipids (Nikolaidis and Mougios, 2004). WAT in mammalian body forms a few depots and can generally be classified into visceral and subcutaneous AT differing in terms of the composition of triacylglycerols-forming FA (Garaulet et al., 2006). Published data about the effects of exercise on FA composition in human AT are limited. An early studies revealed a decrease in oleic acid (18:1) and increase in linoleic acid (18:2 n-6) content in subcutaneous AT after chronic training (Allard et al., 1973; Sutherland et al., 1981). The decrease in the level of 18:1, the main monounsaturated FA (MUFA), which was observed in both studies mentioned above, might be associated with reduced activity of stearoyl-CoA desaturase (SCD1) in AT (Nikolaidis and Mougios, 2004). Since metabolic disorders were shown to be associated with enhanced synthesis of 18:1 and other MUFA by SCD1 (Mika et al., 2015), a post-exercise decrease in AT content of 18:1 may be considered a favorable change. Published evidence suggests that physical training may contribute to a preferential mobilization of some FAs from AT. Already after 2 weeks of the training in senior oarsmen, the authors observed a significant decrease in total serum triacylglycerols and cholesterol, along with changes in the FA profile of AT: a decrease in palmitoleic acid (16:1) and an increase in stearic acid (18:0) content, comparing to previously untrained controls (Danner et al., 1984). A more recent study demonstrated that 6 months of increased physical activity contributed to a significant increase in 18:2 n-6 in overweight elderly subjects, while no such effect was observed in untrained controls (Sjögren et al., 2012). Taken altogether, this sparse evidence from human suggests that chronic exercise may contribute to a decrease in 18:1 content, with concomitant increase in 18:2 n-6 and 18:0. Since 18:1 is the main FA found in triacylglycerols (Ntambi and Miyazaki, 2003; Liu et al., 2011), the decrease in its content may contribute to a relative increase in other FAs. 18:2 n-6 is an essential FA, a substrate for synthesis of other n-6 PUFA, that in turn may be than converted into proinflammatory oxylipins, including eicosanoids (Mika and Sledzinski, 2017). However, regular exercise training seems to reduce systemic inflammation (Görgens et al., 2015). More data in this matter originate from rodent models, and based on this evidence we may compare the effect of exercise on FA composition in various WAT depots, as well as in BAT (May et al., 2017). Most of the animal studies demonstrated that chronic exercise contributed to a decrease in MUFA content, which is consistent with the observations made in humans (Nikolaidis and Mougios, 2004). Regarding polyunsaturated FAs (PUFAs), most animal studies showed an increase in their content, especially n-6 PUFAs; however, in some studies, the post-exercise levels of PUFAs were lower than prior to the exercise or remained unchanged. The chronic exercise-induced changes in PUFA content in AT are depot-specific (Bailey et al., 1993; Nikolaidis and Mougios, 2004). Petridou et al. (2005) reported decrease in MUFA levels after chronic exercise and an increase in n-6 PUFA content in visceral WAT but not in subcutaneous WAT. Among the MUFAs of visceral fat, chronic exercise contributed to a decrease in 16:1, but not in 18:1 (Petridou et al., 2005); the same phenomenon was also observed by Rocha-Rodrigues et al. (2017b) in a rat model. In recent study conducted by May et al. (2017) the authors performed a comprehensive analysis of FA content in phospholipids and triacylglycerols from subcutaneous WAT and BAT of mice subjected to a 3-week exercise training. The study demonstrated that while the exercise contributed to a significant increase in MUFA level and a significant decrease in PUFA content in WAT phospholipids, an inverse phenomenon, i.e., a decrease in MUFAs and an increase in PUFAs was observed in BAT phospholipids. Moreover, a significant decrease in triacylglycerol content of SFAs, MUFAs and PUFAs in BAT and triacylglycerol content of PUFAs in WAT was observed (May et al., 2017). However, it should be stressed that in that study, FA content was expressed in nmol per mg of protein, rather than as a percentage of total FAs as in previously mentioned experiments. These findings suggest that post-exercise changes in FA composition of AT are not only depot- but also lipid molecule-specific. In both WAT and BAT, physical exercise contributed to a significant decrease in the total content of triacylglycerols, but with a concomitant increase in the level of triacylglycerols containing long-chain FAs (58–60 total carbons) (May et al., 2017). The effects of exercise on FA composition in AT and other AT parameters in human and animal studies is summarized in Table 1.

**Table 1:** The summary of effects of exercise on adipose tissue metabolism and adipokine secretion.

The effect of exercise on:		Subcutaneous WAT	Visceral WAT	BAT	Reference
Fatty acid profile	18:0	↑^h^	DNF	DNF	Danner et al., 1984
	16:1	↓^h^	↓^r^	DNF	Danner et al., 1984; Petridou et al., 2005; Rocha-Rodrigues et al., 2017b
	18:1	↓^h^	NC^r^	DNF	Allard et al., 1973; Sutherland et al., 1981; Petridou et al., 2005; Rocha-Rodrigues et al., 2017b
	MUFA	↓^h^, NC or ↓^r^	↓^r^	DNF	Bailey et al., 1993; Nikolaidis and Mougios, 2004; Petridou et al., 2005; Rocha-Rodrigues et al., 2017b
	MUFA in TG	NC^m^	DNF	↓^m^	May et al., 2017
	MUFA in PL	↑^m^	DNF	↓^m^	May et al., 2017
	18:2 n-6	↑^h^, ↑^r^	NC^r^	DNF	Sutherland et al., 1981; Bailey et al., 1993; Sjögren et al., 2012
	n-6 PUFA	↑^h^, ↑^r^	DNF	DNF	Nikolaidis and Mougios, 2004
	PUFA in TG	↓^m^	DNF	↓^m^	May et al., 2017
	PUFA in PL	↓^m^	DNF	↑^m^	May et al., 2017
Expression/activity of enzymes of lipid metabolism	HSL	NC^m^	↑^m^, ↓^r^	DNF	Chen et al., 2015; Holland et al., 2016; Woo and Kang, 2016
	ATGL	NC^m^	↑^m^	DNF	Chen et al., 2015; Woo and Kang, 2016
	SCD1	NC^h^, NC^m^	DNF	↓^m^	Sjögren et al., 2012; May et al., 2017
	ACC	↑^m^	↑^m^, ↓^r^	↓^m^	May et al., 2017; Holland et al., 2016; Rocha-Rodrigues et al., 2017a
	FADS1	DNF	↑^r^	DNF	Rocha-Rodrigues et al., 2017a
	ELOVL5	DNF	↑^r^	DNF	Rocha-Rodrigues et al., 2017a
Expression/ secretion of adipokines	Adiponectin	NC^r^, ggg or NC^h^	↑^r^, ↑^h^	DNF	Görgens et al., 2015; Kato et al., 2018
	Leptin	NC^h^	DNF	DNF	Görgens et al., 2015
	IL-6	↓ or NC^h^	DNF	DNF	Bruun et al., 2006; Klimcakova et al., 2006
	Apelin	DNF	↑^r^	DNF	Kazemi and Zahediasl, 2018
Adipose tissue beiging		↑^r^, ↑^m^	DNF	–	Lehnig and Stanford, 2018

*WAT, white adipose tissue; BAT, brown adipose tissue; ggg, increased; ↓, decreased; NC, not changed significantly; ^*h*^, in human; ^*r*^, in rat; ^*m*^, in mouse; DNF, data not found; TG, triacylglycerols; PL phospholipids; HSL, hormone-sensitive lipase; ATGL, adipose triglyceride lipase; SCD1, stearoyl-CoA desaturase 1; ACC, acetyl-CoA carboxylase; FADS1, fatty acid desaturase 1; ELOVL5, fatty acid elongase 1; Il6, interleukin 6*.

## Effect of Exercise on FA Metabolism in AT

The post-exercise decrease in triacylglycerols content in AT is with no doubt a consequence of enhanced lipolysis. The process, initiated by adipose triglyceride lipase (ATGL), is then continued by hormone-sensitive lipase (HSL), upon phosphorylation thereof; eventually, the last FA chain is hydrolyzed by monoacylglycerol lipase (MAGL) (Chen et al., 2015). While the rate of lipolysis is decreased by obesity and high-fat diet, chronic exercise was shown to normalize the markers of this process, phosphorylated HSL and ATGL, in mice that have been previously maintained on a high-fat diet (Chen et al., 2015). Surprisingly, however, Holland et al. (2016) demonstrated that voluntary wheel running for 42 days contributed to a decrease in phosphorylated HSL level in rats. In contrast, chronic exercise was shown to stimulate the activity of lipolytic enzymes in the adipocytes of obese mice (Woo and Kang, 2016), and a recent study demonstrated that endurance exercise contributed to an increase in triacylglycerol lipase activity in human AT, especially during the first 10 min of the training (Petridou et al., 2017). An upregulation of HSL after chronic exercise was also mentioned in a review paper published by Steinberg (2009). Moreover, irisin, an adipokine released by working muscles, was shown to induce the expression of ATGL and HSL in 3T3L1 adipocytes (Gao et al., 2016). Thus, the results of most published studies suggest that physical exercise may stimulate lipolytic activity within AT, that may contribute to more efficient reduction of AT mass and/or prevent accumulation thereof.

Physical activity may also modulate FA synthesis, desaturation and elongation. The reduction of MUFA content after chronic exercise reported by many authors might be a consequence of a decrease in FA desaturation by SCD1 (Nikolaidis and Mougios, 2004). However, this conclusion is based on the desaturation indices calculated from SFA and MUFA contents in AT. Thus, it cannot be excluded that those parameters were also influenced by preferential uptake and release of certain FAs in AT during exercise (Halliwell et al., 1996). One study demonstrated that chronic exercise did not affect the expression of SCD1 in mice subcutaneous WAT, but contributed to lesser activity of this enzyme in BAT (May et al., 2017). Also in human subcutaneous AT, the expression of SCD1 gene remained unchanged after the chronic exercise (Sjögren et al., 2012). Published data about the exercise-induced changes in the activity of other lipogenic enzymes are inconclusive. According to May et al. (2017), 3-week exercise contributed to an increase in acetyl-CoA carboxylase (ACC) mRNA level in mice subcutaneous WAT, but not in BAT whereby mRNA level for this enzyme was reduced. Similarly, a 6-week exercise resulted in an increase in ACC protein level in visceral WAT of rats (Holland et al., 2016). In contrast, Rocha-Rodrigues et al. (2017a) found reduced activity of ACC in visceral AT of rats subjected to an 8-week endurance training. The same study demonstrated a post-exercise increase in the expressions of enzymes involved in PUFA metabolism, FA desaturase 1 and elongase 5 (Rocha-Rodrigues et al., 2017a); these findings are consistent with the results published by other authors who observed a chronic exercise-induced increase in PUFA content and elongase indices (Nikolaidis and Mougios, 2004). In line with those findings, May et al. (2017) found elevated levels of mRNA for elongase 3 and 4 in AT from chronically exercised mice. Taken altogether, the abovementioned findings suggest that the effect of exercise on the expression of enzymes involved in lipid metabolism may vary depending on FA group and AT depot.

## Impact of Exercise on Adipokine Secretion in AT

Muscle work during the exercise may activate a signaling cascade; specifically, myokines released from the muscle cells may trigger a release of adipokines, signaling molecules synthesized in the AT. Aside from the production of adipokines, AT can also synthesize many myokines, among others IL-6, MCP1, TNFα, visfatin and myostatin, which are collectively referred to as adipomyokines (Görgens et al., 2015). Thus, plasma level of adipomyokines does not necessarily reflect solely the pool which is synthesized in the AT and acts on the muscles, and the origin of each molecule should be identified at a cellular level. Adiponectin is an insulin-sensitizing hormone that enhances FA oxidation in the muscles and downregulates the synthesis of lipids and glucose in the liver (Swierczynski and Sledzinski, 2012). The evidence from both human and animal studies analyzing the effects of exercise on serum adiponectin level is inconclusive; chronic exercise was either shown to increase the serum concentration or expression in AT of this adipokine or did not affect it at all (Kato et al., 2018; Lehnig and Stanford, 2018). Available data imply that the release of adiponectin from human AT may depend on exercise intensity (Görgens et al., 2015). Another adipokine, leptin, is synthesized primarily in the AT, regulates appetite and boosts peripheral metabolism (Swierczynski and Sledzinski, 2012). Chronic exercise was shown to contribute to a decrease in serum leptin concentration, but this effect was associated with the reduction of AT mass (Lehnig and Stanford, 2018). However, previous studies demonstrated that excess body weight was associated with leptin resistance (Swierczynski and Sledzinski, 2012), and chronic exercise might improve leptin sensitivity (Kang et al., 2013). Thus, the simultaneous reduction of serum leptin level and AT mass does not necessarily correspond to a decrease in the activity of this adipokine. Serum concentration of IL-6, acting as an anti-inflammatory myokine, was shown to increase substantially after acute exercise, and this effect was demonstrated to result from local synthesis of IL-6 by the working muscles. However, the level of IL-6 after chronic exercise was decreased or remained unchanged (Görgens et al., 2015). Also, the expression of IL-6 in the AT did not change or was reduced, depending on the type of chronic exercise (Bruun et al., 2006; Klimcakova et al., 2006). Generally, moderate chronic exercise seems to be associated with a decrease in the release of pro-inflammatory cytokines, such as TNF-α, leptin and MCP-1, from the AT and working muscles; this may contribute to attenuation of systemic inflammation (Görgens et al., 2015). A recent study demonstrated that chronically exercised rats showed enhanced expression of apelin, an adipomyokine that decreases insulin resistance (Kazemi and Zahediasl, 2018). Apelin induced glucose uptake by AT, but at the same time decreases triglyceride amounts in mouse AT and lipid storage in 3T3-L1 preadipocytes (Indrakusuma et al., 2015). Also, serum concentration of resistin, an adipokine promoting insulin resistance, was shown to decrease in rats subjected to an chronic exercise (Shirvani and Arabzadeh, 2018). Altogether, these findings suggest that chronic exercise may improve the profile of adipokines released from the AT, and thus, may be beneficial for health.

## Exercise Leads to at “Beiging” — A Process Mediated by Myokines

Aside from the metabolic processes discussed above (lipolysis, FA uptake, FA synthesis), FAs are also oxidized in mitochondria, in a process referred to as β-oxidation. A number of previous studies demonstrated that chronic exercise enhanced mitochondrial activity in visceral and subcutaneous AT, in both rodents (Stallknecht et al., 1991; Sutherland et al., 2009; Vernochet et al., 2012; Wu et al., 2012; Stanford et al., 2015a,b) and humans (Ruschke et al., 2010; Rönn et al., 2014). The process of mitochondrial β-oxidation in the AT is not as intensive as in the muscles but still can provide an extra pool of energy for adipocytes after the exercise. Furthermore, there is one AT depot that shows greater mitochondrial activity than visceral and subcutaneous WAT; this is BAT which contains numerous mitochondria whereby FAs undergo oxidization, becoming a source of energy for thermogenesis. The main protein involved in thermogenesis is uncoupling protein 1 (UCP1), mediating proton leakage across the inner mitochondrial membrane into the mitochondrial matrix, and thus, playing a role in heat production (Lehnig and Stanford, 2018). Recent research showed that chronic exercise may contribute to “beiging” of subcutaneous WAT, a process which is also referred to as the “browning” of AT. During the process of “beiging,” a phenotype and metabolism of white adipocytes in the AT change and resemble the respective characteristics of brown adipocytes in the BAT (Lehnig and Stanford, 2018). This phenotypic and functional switch includes an increase in mitochondrial respiration and enhancement of UCP1 protein expression, as well as the upregulation of other genes characteristic for BAT (Wu et al., 2012). In one study, ablation of beige adipocytes resulted in the development of obesity and insulin resistance in mice; this implies that these cells may play a role in the regulation of systemic energy metabolism (Stanford and Goodyear, 2016). While the exercise-induced adipocyte “beiging” has been well documented in rodents, still little is known about this phenomenon in humans (Lehnig and Stanford, 2018). However, it needs to be stressed that BAT in adult humans resembles murine beige AT, rather than the BAT (Wu et al., 2012). A number of potential mechanisms responsible for AT “beiging” have been proposed thus far. According to one hypothesis, the process may be mediated by myokines and small molecules released from working muscles (Lehnig and Stanford, 2018), specifically by irisin (Boström et al., 2012), myostatin (Feldman et al., 2006), meteorin-like 1 (Metrnl) (Rao et al., 2014), lactate (Carriere et al., 2014) and/or β-aminoisobutyric acid (BAIBA) (Roberts et al., 2014).

## Conclusion

Physical exercise stimulates lipolysis, decreases FA uptake by the adipocytes, exerts an effect on FA composition within the AT and modulates the expression of enzymes involved in FA synthesis, elongation and desaturation. Moreover, exercise promotes “beiging” of AT and contributes to an increase in mitochondrial activity, which leads to enhanced FA oxidation in the AT. As a result of all those metabolic processes, physically active persons can maintain adequate volume of AT. Chronic exercise influences the release of adipokines, which may attenuate systemic inflammation and prevent insulin resistance. Moreover, transplantation of AT from trained to untrained mice was shown to improve glucose tolerance (Stanford et al., 2015b). Taken altogether, these findings imply that the exercise-induced changes in AT metabolism may exert a beneficial effect on global metabolic health.

## Author Contributions

AM, FM, RB, VD, and TS studied the literature and wrote the manuscript. All authors accepted the final version of manuscript.

## Conflict of Interest Statement

The authors declare that the research was conducted in the absence of any commercial or financial relationships that could be construed as a potential conflict of interest. The handling Editor is currently co-organizing a Research Topic with one of the authors VD, and confirms the absence of any other collaboration.
